# The magnifying effect of a thin shallow stiff layer on Love waves as revealed by multi-component analysis of surface waves

**DOI:** 10.1038/s41598-020-66070-1

**Published:** 2020-06-03

**Authors:** Giancarlo Dal Moro

**Affiliations:** Institute of Rock Structure and Mechanics, Academy of Sciences of the Czech Republic, Prague, Czech Republic

**Keywords:** Geophysics, Seismology

## Abstract

In the last decades, surface wave analysis has become a standard tool for an increasingly large number of geotechnical applications that require the determination of the subsurface shear-wave velocity (V_S_) profile. In the present paper, we investigate the role of a shallow stiff layer on Rayleigh and Love wave propagation. Multi-component synthetic and field data are considered to analyse the vertical (Z) and radial (R) components of Rayleigh waves as well as Love waves (T component). Velocity spectra are analysed according to the *Full Velocity Spectrum* (FVS) approach together with the Rayleigh-wave Particle Motion (RPM) frequency-offset surface that reveals the actual prograde-retrograde motion of Rayleigh waves. The FVS approach to surface wave analysis reveals particularly powerful in case we intend to reproduce the actual modal energy and when, because of complex mode excitation, the velocity spectra cannot be easily interpreted in terms of modal dispersion curves. The analysis of both synthetic and field data highlights two major facts. On one side, along the T component (Love waves) the presence of a thin shallow stiff layer excites higher modes whose top velocity is controlled by the shear-wave velocity of the deeper layers. On the other side, such a stiff layer does not massively influence the velocity spectra of the Z and R components (Rayleigh waves) and the related RPM: irrespective of the presence of the superficial stiff layer, RPM clearly shows the change from retrograde to prograde due to the V_S_ increase in the deep layers. In case a superficial stiff layer is present (this condition is quite common in urbanized areas such as the one of the field dataset here considered), Love waves can be then an interesting tool for an expeditious estimation of the V_S_ of the deep layers.

## Introduction

Surface wave analysis is routinely performed for a number of applications aimed at depicting the vertical shear-wave velocity (V_S_) profile in classical crustal studies^1–7^, as well as in geotechnical and seismic-hazard applications aimed at characterizing the first tens of meters and estimate possible amplification phenomena^8–13^.

In order to retrieve the dispersive properties, several techniques can be adopted based on the specific needs, site characteristics and goals. Techniques are usually grouped into two main families: active (e.g. Multichannel Analysis of Surface Waves - MASW, Holistic analysis of Surface waves - HS; Frequency Time Analysis – FTAN^1,14–19^) and passive (e.g. *f-k* analysis, Spatial AutoCorrelation - SPAC, Extended Spatial AutoCorrelation - ESAC and Miniature Array Analysis of Microtremors^20–23^).

Since most of the standard methods commonly adopted are based on just one component and rely on the analysis of the propagation velocities only, the results are necessarily prone to non-uniqueness of the solution and interpretative issues^18,19,24–27^.

As a matter of fact, any surface non-invasive methodology inevitably suffers from non-uniqueness problems since different subsurface models can equally explain the observed data^2,18,28^.

The joint analysis of different and complementary observables represents an effective approach to this well-known problem^13,19,25,29–31^. While the analysis of one single component furnishes a subsurface model with a certain ambiguity/uncertainty, by jointly analysing two or more observables the ambiguity of the solution is significantly reduced: the larger the number of independent observables we include in the joint analysis, the smaller the ambiguity (i.e. the higher the reliability of the retrieved model).

For instance, while considering Rayleigh waves, the acquisition of both the vertical (Z) and radial (R) components allows also the computation and analysis of the Radial-to-Vertical Spectral Ratio (RVSR) and the Rayleigh-wave Particle Motion (RPM) data which can be jointly inverted with the Z and R velocity spectra^13,19,29,32^.

Joint inversion can be accomplished considering the single objective function defined as the summation of the misfits obtained from the considered observables^33^ or through a multi-objective inversion scheme ^13,19,27,31,34–38^.

Several recent studies^17,25,27,39–42^ have pointed out the utility of Love waves in near-surface applications. It was shown that while Rayleigh waves can be extremely complex to interpret in terms of modal dispersion curves, Love-wave velocity spectra are usually much simpler and therefore represent an important tool to avoid pitfalls in the interpretation of the Rayleigh-wave velocity spectra.

Furthermore, in some previous studies about Love waves^18,40^, it was observed that a superficial stiff layer may excite higher modes and give rise to peculiar phase-velocity spectra.

A stiff layer can be considered as the opposite of a Low-Velocity Layer (LVL), i.e. as a layer whose velocity is higher than the velocities of the layers above and below it (the case of a superficial layer is just a special case).

In the present paper, the effect of a shallow stiff layer is investigated in detail through the analysis of the behaviour of Rayleigh and Love waves for a series of synthetic and field datasets.

In general terms, it must be underlined that higher modes do not represent a problem but rather a source of valuable information that, if properly handled, significantly helps in better constraining the inversion process, thus the retrieved subsurface model^13,17,18,25,40^.

Data and analyses presented in this paper refer to multi-channel, multi-offset and multi-component data that can be generically referred to as MASW (Multichannel Analysis of Surface Waves), although the presented analyses go beyond the classical inversion of the modal dispersion curve(s) interpreted from the phase-velocity spectrum obtained from a single-component dataset and attempt to describe and analyse the propagation of surface waves in a more comprehensive (holistic) perspective.

Surface-wave dispersion is here analysed according to the *Full Velocity Spectrum* (FVS) approach^13,18,19,25,26^, thus without interpreting the velocity spectra in terms of dispersion curves. Furthermore, Rayleigh waves are analysed not only with respect to the velocity spectra of the vertical and radial components, but also considering the actual particle motion.

The Rayleigh-wave Particle Motion (RPM) frequency-offset surface^19,32^ was recently introduced in order to provide a quantitative description of the actual Rayleigh-wave motion, which is often far from being retrograde^43,44^ (as often erroneously believed). Such a surface represents the correlation values between the radial component and the Hilbert transform of the vertical component as a function of both the frequency and offset: +1 indicates a pure retrograde motion, while −1 a pure prograde motion. Since the actual particle motion is a complex mix, the correlation values range between +1 and −1 as a function of both the frequency and offset.

In order to better constrain the subsurface V_S_ model, field data are therefore analysed by considering the RPM frequency-offset surface jointly with the phase velocity spectra of all the three considered components (Z, R and T)^13,19,29^. The nomenclature adopted is described in details in several papers^18,25,26,45,46^: the acronyms ZVF and RVF refer to the vertical (Z) and radial (R) components while considering a Vertical Force (VF) source (e.g. a common sledgehammer or weight drop), while THF is the transversal (T) component when a Horizontal Force (HF) is applied.

## Synthetic data: the phase-velocity spectra

**Figure 1:**
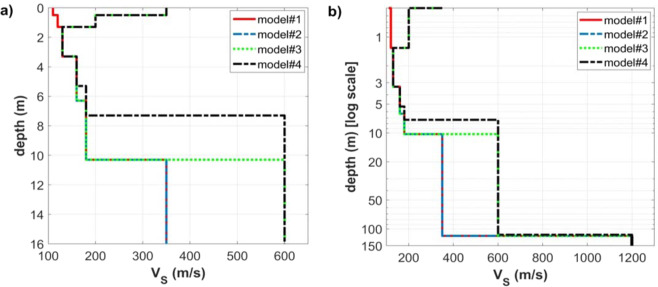
The four models considered for the synthetic data presented in Figs. 2–7: (**a**) V_S_ profile down to 16 m (linear scale); (**b**) V_S_ profiles down to 150 m (logarithmic scale used to emphasize the shallow layers but showing also the deep bedrock, that cannot be sensed while using short arrays as the ones considered in the present work).

In order to investigate the role of a surficial stiff layer on surface-wave propagation, we computed a series of synthetic seismograms based on the modal summation approach^45–49^ [we considered the first 10 modes]. The four considered models (Fig. 1) differ because of the presence of a shallow stiff layer (models #2, 3 and 4), the depth of the “deep” (gravel-like) layer (for the models#1, 2 and 3 is slightly more than 10 m while for the model#4 slightly more than 7 m) and for the V_S_ value of the deep stiff material (350 m/s for the models#1 and 2 and 600 m/s for the models#3 and 4).

The effect of a thin superficial stiff layer can be highlighted through the comparison of the seismic traces and phase-velocity spectra of the four considered models.

**Figure 2:**
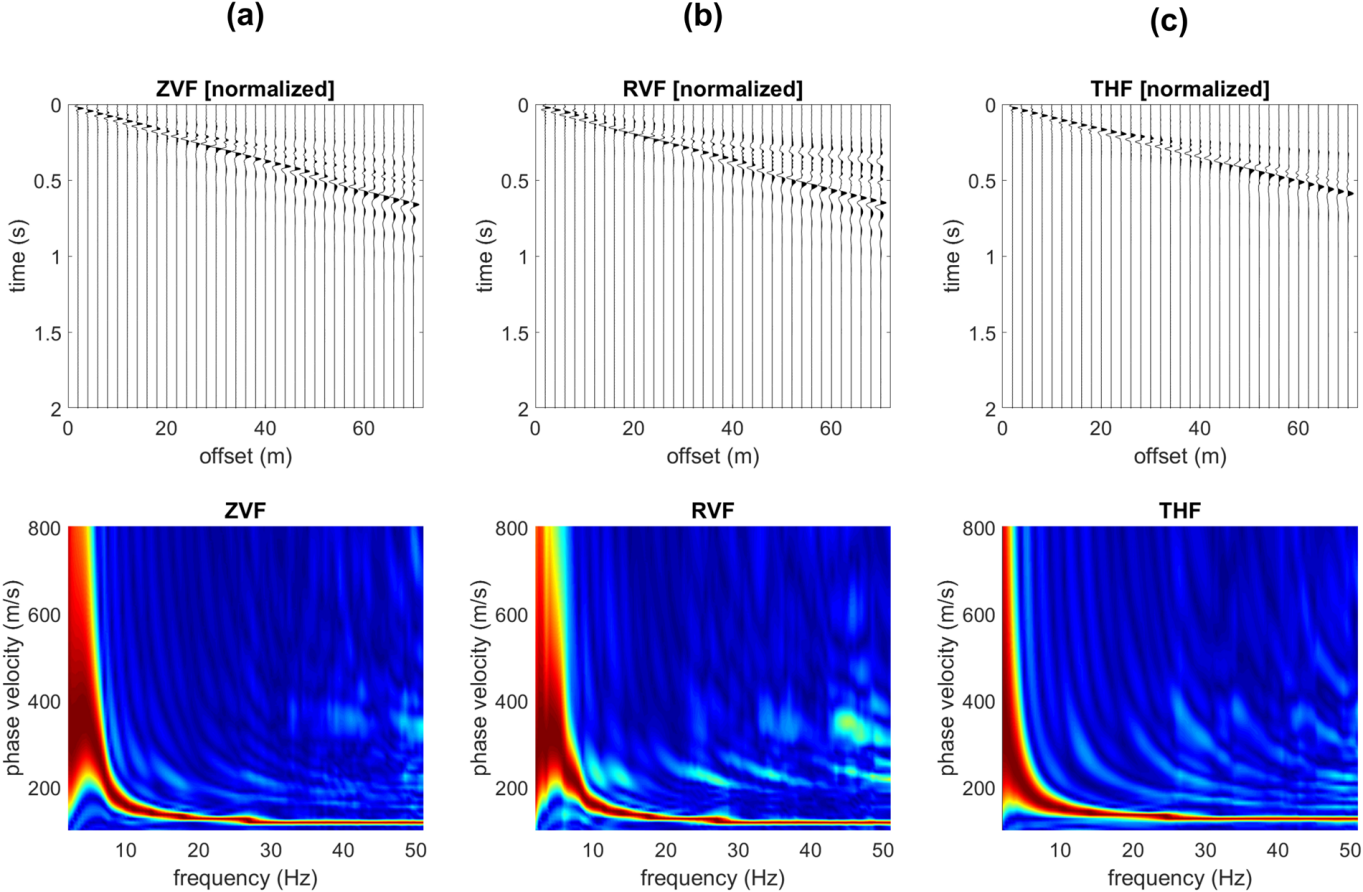
Synthetic seismograms and phase-velocity spectra for the model#1 (no superficial stiff layer): (**a**) ZVF (vertical component of Rayleigh waves considering a Vertical Force source); (**b**) RVF (radial component of Rayleigh waves); (**c**) THF (Transversal component obtained considering a Horizontal Force source - Love waves).

**Figure 3:**
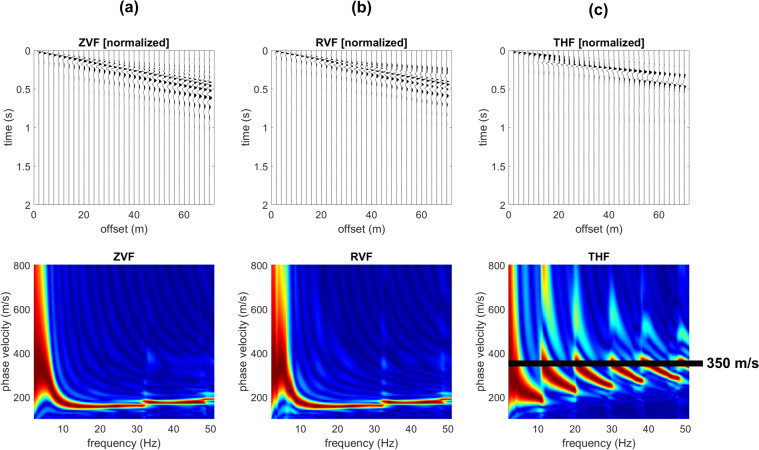
Effect of a superficial stiff layer: synthetic seismograms and phase velocity spectra for the model#2 (which differs from model#1 only because of a thin surficial stiff layer): (**a**) ZVF (vertical component of Rayleigh waves); (**b**) RVF (radial component of Rayleigh waves); (**c**) THF (Love waves). The top velocity of the THF higher modes is closely related to the V_S_ of the deep layer (see model#2 in Fig. 1).

While Rayleigh-wave velocity spectra show minor differences and just in the very high frequency range (compare for instance the Z component in Figs. 2a and 3a), the effect of the shallow stiff layer on Love waves is definitely more significant. In fact, when a shallow stiff layer is present, large-amplitude higher modes appear (compare Figs. 2c and 3c) and their top velocities closely relate to the V_S_ values of the deep (gravel-like) layer.

**Figure 4:**
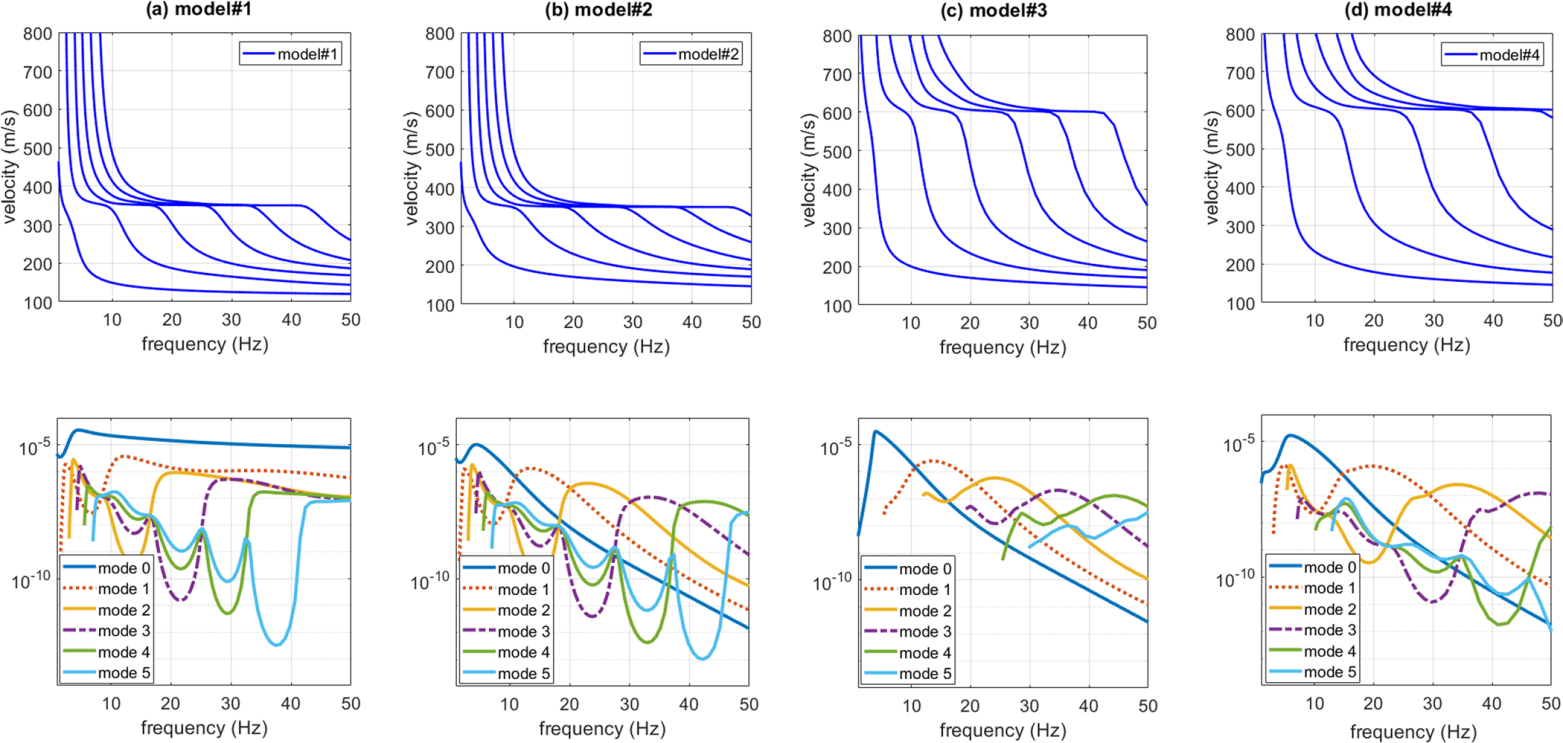
Transversal (T) component (Love waves): phase-velocity modal dispersion curves (upper panel) and power spectra (lower panel) for the four considered models (model#1 is the one without the surficial stiff layer). Shown the curves for the first six modes. The power spectra shown in the lower panel provide the evidence of the reason why, in case a shallow stiff layer is present, higher modes dominate over the fundamental one. Further comments in the text.

This is apparent also by comparing the theoretical power spectra^50^ of the transversal component (Love waves) for the four considered models. The computed power spectra express the amount of energy of each mode and are reported in Fig. 4 together with the respective modal dispersion curves. By comparing the power spectra of the four models it is clear that the presence of a shallow stiff layer excites the higher modes that have otherwise a lower energy compared to the fundamental mode (Fig. 4a). For the model#2, for instance, between about 30 and 39 Hz the third higher mode is the most energetic and the top velocity of the respective modal dispersion curve is about 350 m/s (compare the phase-velocity spectra reported in Fig. 3c and the power spectra in Fig. 4b).

**Figure 5:**
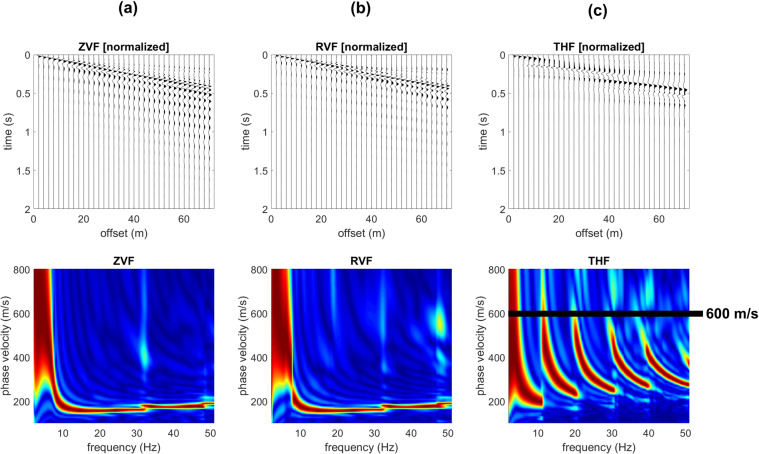
Effect of a superficial stiff layer: synthetic seismograms and phase velocity spectra for the model#3: (**a**) ZVF (vertical component of Rayleigh waves); (**b**) RVF (radial component of Rayleigh waves); (**c**) THF (Love waves). The top velocity of the THF higher modes is closely related to the V_S_ of the deep layer (see model#3 in Fig. 1).

In Fig. 5 are shown the synthetic traces and phase-velocity spectra for the third model (Fig. 1). With respect to the model considered in Fig. 2 now the V_S_ value of the deep layer is increased to 600 m/s (while previously was 350 m/s). As apparent from the Love-wave velocity spectrum reported in Fig. 5c, now the top velocities of the higher modes is larger (about 600 m/s). On the other hand, Rayleigh waves (Fig. 5a,b) show an increase in the energy associated to the higher modes just in the very high frequency range and for the phase velocities at frequencies lower than about 9 Hz (associated to the deep layer).

It is once again clear that the top velocities of the Love-wave higher modes are closely related to the shear-wave velocity of the deep layer even at very high frequencies while Rayleigh waves provide information about the deep layers just in the low-frequency range.

The power spectra of the model#3 (Fig. 4c) show, for instance, that in the 21–31 Hz frequency range the most energetic mode is the second higher mode that reaches a “top velocity” of about 600 m/s (see the phase-velocity spectrum in Fig. 5c).

Is it clear that the top velocities observed in the phase-velocity spectra (computed from the seismic traces according to the phase-shift method^14^) correspond to the area (velocities) where Love-wave modal dispersion curves reach a plateau value of about 350 m/s for the models #1 and 2 and 600 m/s for the models #3 and 4 (see modal dispersion curves in the upper plots of Fig. 4).

**Figure 6:**
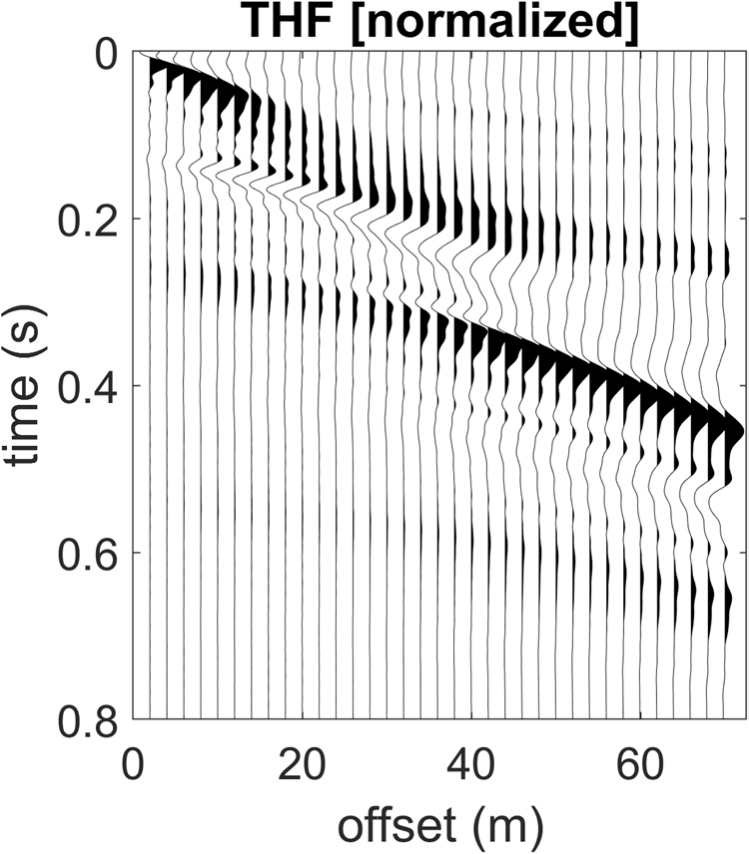
Close up of the THF (Love waves) normalized traces shown in Fig. 5c (model#3). While recording Love waves, the characteristic hyperbola-like trend is a typical feature revealing the presence of a shallow stiff layer (compare with Fig. 2c).

We can also point out that the presence of a shallow stiff layer is responsible for a peculiar feature of the seismic traces of the transversal component (Love waves), which assume a hyperbola-like characteristic trend (see Fig. 6 and compare with Fig. 2c).

**Figure 7:**
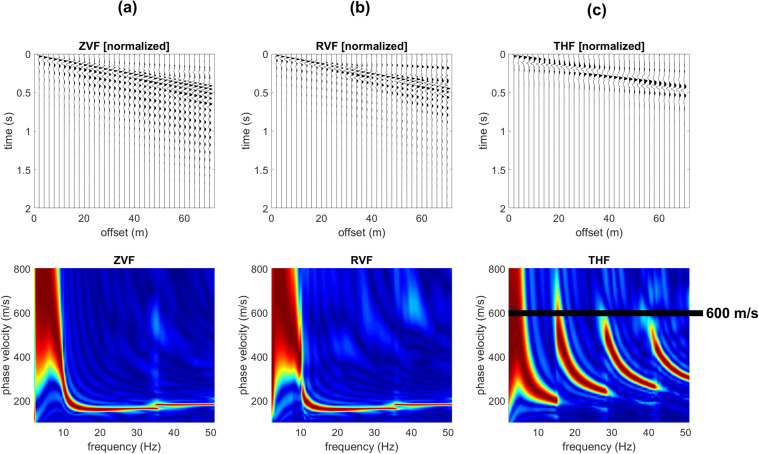
Effect of a superficial stiff layer: synthetic seismograms and phase velocity spectra for the model#4: (**a**) ZVF (vertical component of Rayleigh waves); (**b**) RVF (radial component of Rayleigh waves); (**c**) THF (Love waves). The top velocity of the THF higher modes is closely related to the V_S_ of the deep layer (see model#4 in Fig. 1).

By comparing the data reported in Figs. 5c and 7c (models #3 and 4), it is also clear that the number of higher modes in a fixed frequency range depends on the depth of the deep stiff layer (the shallower the contact, the lower the number of higher modes) but the top velocity of the higher modes does not significantly change.

### Further insights from synthetic data

Data and analyses presented in the previous section demonstrate the effect of a superficial stiff layer on the phase-velocity spectra of Love and Rayleigh waves. While Love waves reveal the deep V_S_ values through their massive higher modes (even at high frequencies), the phase-velocity spectra of Rayleigh waves do not appear dramatically influenced by such a superficial stratigraphic feature.

We might anyway wonder whether the particle motion induced by the Rayleigh waves is or not influenced by the presence of such a shallow stiff layer.

In order to address this point, we can compare the RPM frequency-offset surfaces for model#3 and model#1, i.e. for two models that differ for the presence of a superficial stiff layer.

**Figure 8:**
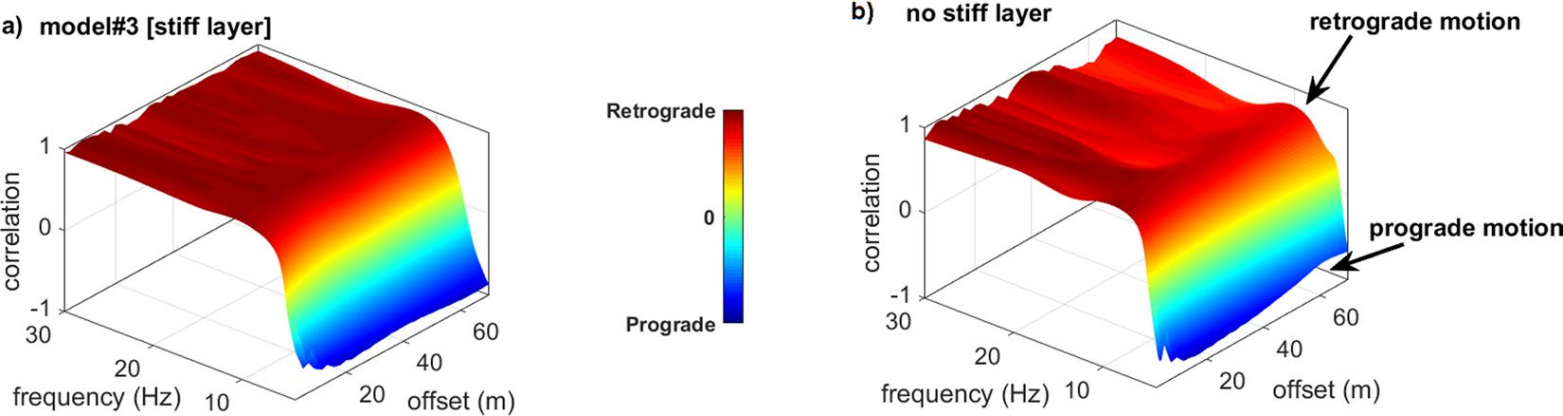
Effect of a stiff superficial layer on the RPM surface: (**a**) RPM surface for the model#3 (with the stiff layer); (**b**) RPM surface of the model without any surficial stiff layer. Between about 4 and 5 Hz, the Rayleigh-wave particle motion changes from retrograde to prograde because of the stiff gravel-like layer at a depth of about 10 m (see Fig. 1) and this phenomenon occurs independently on the presence or not of a superficial stiff layer.

From the comparison of the RPM surfaces shown in Fig. 8a,b, it is clear that in the frequency range of primary interest in common near-surface applications (about 4–30 Hz) the presence of a surficial stiff layer does not significantly affect the prograde-retrograde motion of Rayleigh waves (between 4 and 5 Hz Rayleigh waves change their motion from retrograde to prograde because of the large V_S_ increase at about 10 m of depth).

As shown in the previous section, Love waves can be quite effective for the characterization of even relatively-deep features but a further question arises: what is the influence of the array length?

**Figure 9:**
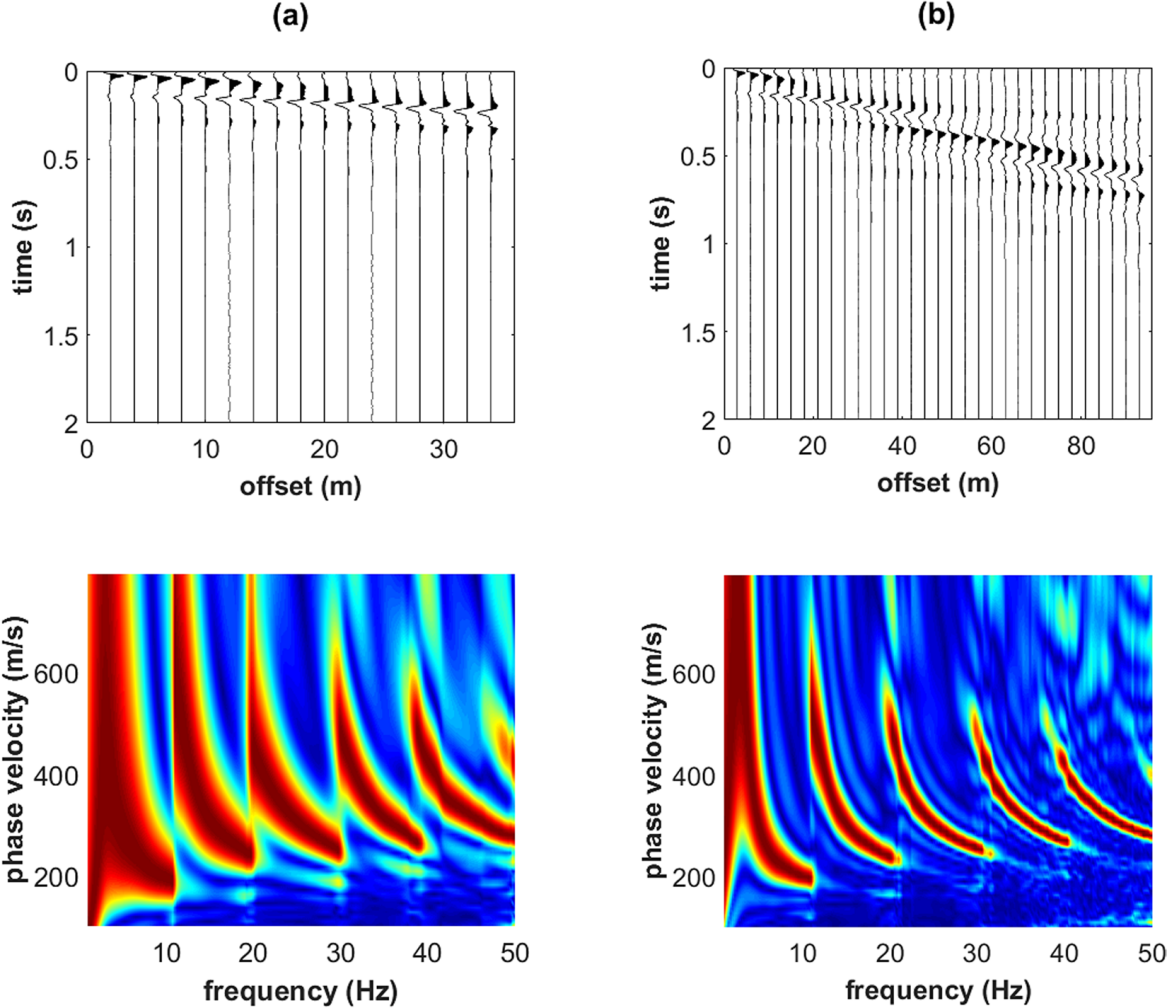
THF (Love waves) synthetic data for the model#3 (Fig. 1) while considering two different arrays: (**a**) synthetic traces and phase velocity spectrum for a short array; (**b**) synthetic traces and phase velocity spectrum for a longer array (compare also with Fig. 5c, where a different array is considered).

In order to briefly investigate this point, two final synthetic datasets were computed considering the model#3 (Fig. 1). The synthetic traces and velocity spectra obtained while considering two different arrays are reported in Fig. 9. The comparison of the computed phase-velocity spectra shows that, in a given frequency range, the number of excited higher modes is the same. On the other side, because of simple mathematical facts (the velocity spectra shown in this work were computed according to the phase-shift method^14^), in case of very short arrays the velocity spectrum is less focused.

### Surface-wave dispersion analysis: the FVS approach in brief

In order to clarify the analysis of the field dataset presented in the next section, we here briefly summarize the *Full Velocity Spectrum* (FVS) approach to dispersion analysis^13,18,19,25,26^. In fact, the analysis of interpreted modal dispersion curves^18,27,51^ is not the only way to analyse surface wave propagation and the FVS technique represents a possible improved approach.

The FVS approach is based on the computation of the synthetic traces hereby accomplished via modal summation^45,46,48^.

Considering the simple single-component case, once the velocity spectrum of the field traces is computed, the FVS inversion consists of three main steps:

1. computation of the synthetic trace(s) of a tentative model;
2. computation of the velocity spectra of the synthetic traces;
3. computation of the misfit between the velocity spectra of the field and synthetic traces.

**Figure 10:**
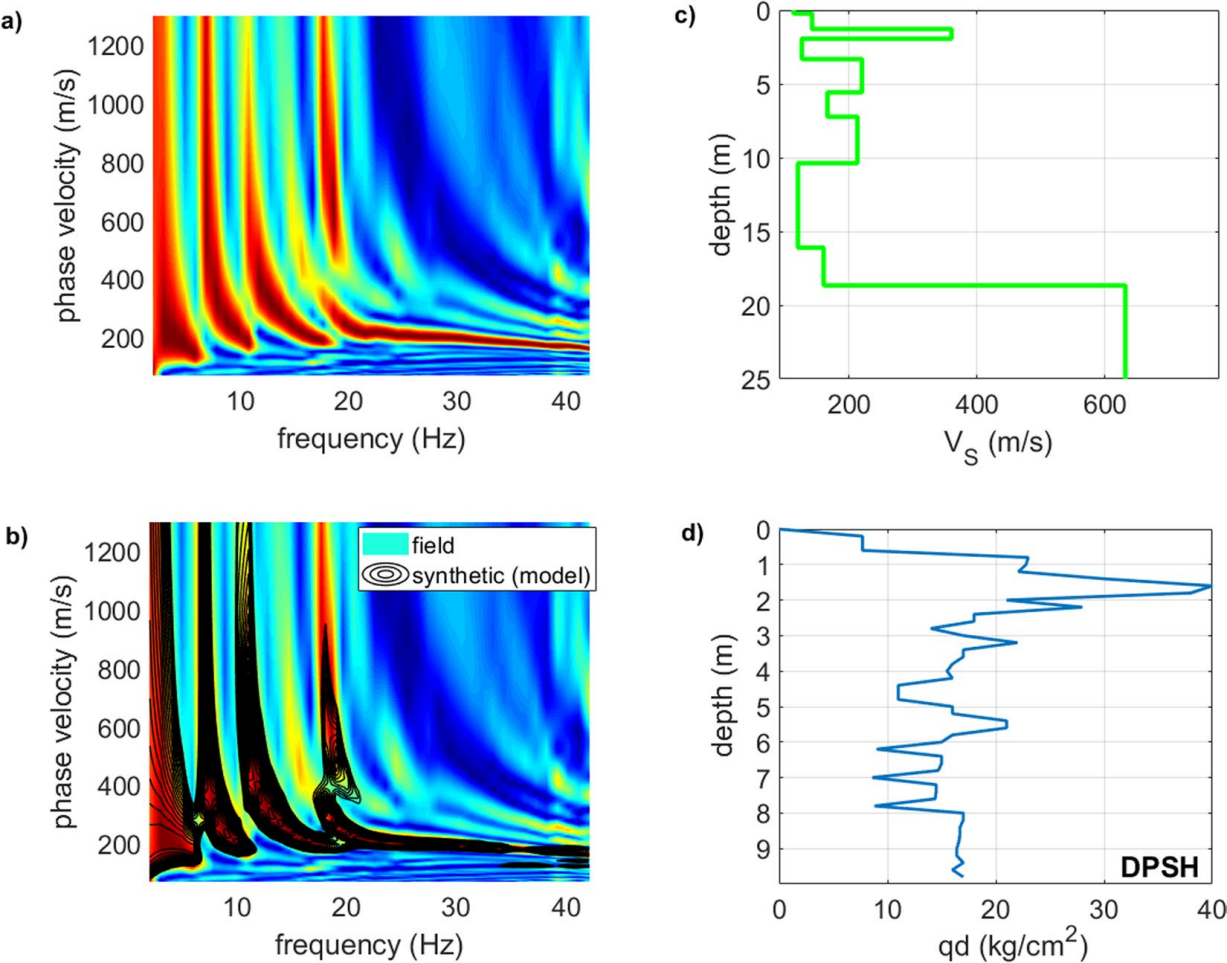
Example of single-component FVS analysis: (**a**) phase-velocity spectrum of a field dataset (THF component – i.e. Love waves); (**b**) phase-velocity spectra for the field data (background colours) and for the model obtained through the FVS inversion (overlaying black contour lines - the agreement between the two velocity spectra is apparent); (**c**) identified V_S_ model; (**d**) qd values (dynamic point resistance) from a DPSH (Dynamic Probing Super Heavy) penetrometer test performed down to 9.8 m. The shallow stiff layer at a depth of about 2 m is responsible for the higher modes that largely dominate the THF velocity spectrum.

These three steps are implemented within a heuristic optimization algorithm that minimizes the misfit, thus eventually providing a subsurface model that has a velocity spectrum as close as possible to the velocity spectrum of the field data. It is important to understand that this way we deal with the entire velocity spectrum (i.e., the frequency-velocity matrix) and not with a dispersion curve (i.e., a frequency-velocity curve that represents a personal - i.e. subjective - interpretation of the velocity spectrum in terms of modal dispersion curves). Figure 10 reports an example of single-component FVS analysis and intends to briefly and visually express how, during a FVS inversion process, we aim at identifying a subsurface model whose velocity spectrum is as close as possible to the one of the field traces. In fact, as Fig. 10b clearly shows, the phase-velocity spectrum (black contour lines) of the synthetic traces of the subsurface model identified by means of the above-mentioned inversion scheme matches quite well with the velocity spectrum of the field data (background colours and Fig. 10a).

**Figure 11:**
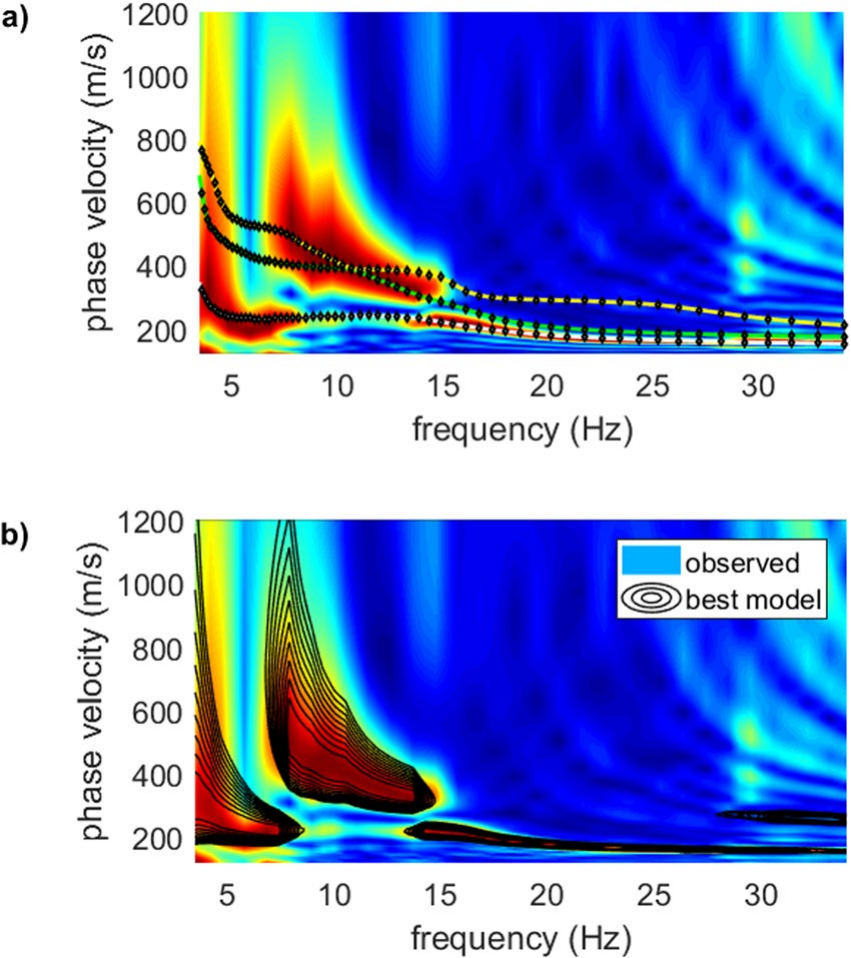
Upper plot: standard surface-wave analysis via modal dispersion curves (background colours represent the phase-velocity spectrum of a field dataset while the three overlying dispersion curves refer to the first three modes of a tentative subsurface model); lower plot: FVS analysis of surface-wave dispersion: the overlaying black contour lines refer to the phase-velocity spectrum of the model identified via FVS inversion. The field and synthetic velocity spectra are in apparent good agreement. See text for comments.

We should highlight that the classical analysis of the modal dispersion curves do not demonstrate that a certain mode is (or not) excited. On the other side, the FVS approach provides the evidence that a certain mode is actually excited and we can therefore better constrain the inversion process. The analyses reported in Fig. 11 can help to further clarify this point. In the upper plot (Fig. 11a) we show an example of standard modelling based on the modal dispersion curves. The velocity spectrum of the field data (background colours) is interpreted so that the energy below 7 and above 15 Hz pertains to the fundamental mode while in between to higher modes. Anyway, this standard approach to surface-wave analysis via modal dispersion curves presents a clear problem: modal curves do not show which modes are actually exited. In fact, considering the data reported in Fig. 11a we might ask: how can we provide the proof that between 7 and 15 15 Hz higher modes are actually excited? Or, similarly: why below 6 Hz and between 15 and 30 Hz the data are dominated by the fundamental mode and not by higher overtones? How can we demonstrate that, in a given frequency range, the considered subsurface model actually excites certain modes?

The FVS approach provides the evidence (i.e. the proof) that, frequency by frequency, certain modes are (or not) excited.

In fact, if we consider the data reported in Fig. 11b, we can see that the velocity spectrum of the identified model (overlying black contour lines) excellently reproduces the velocity spectrum of the field data. In other terms, the identified model is associated to a velocity spectrum that, in the 7–15 Hz frequency range, is actually dominated by higher modes while outside that frequency range the fundamental one dominates.

Furthermore, the FVS approach goes beyond the subjectivity of the classical modal dispersion curves which are picked based on the personal understating of the experimental velocity spectra and can therefore be wrong^18,24^.

For the analysis of the field dataset presented in the next section, we considered multi-component data that enable us to better constrain the inversion procedure and overcome the non-uniqueness of the solution^13,18,19,25,26,30^.

### A field dataset

**Figure 12:**
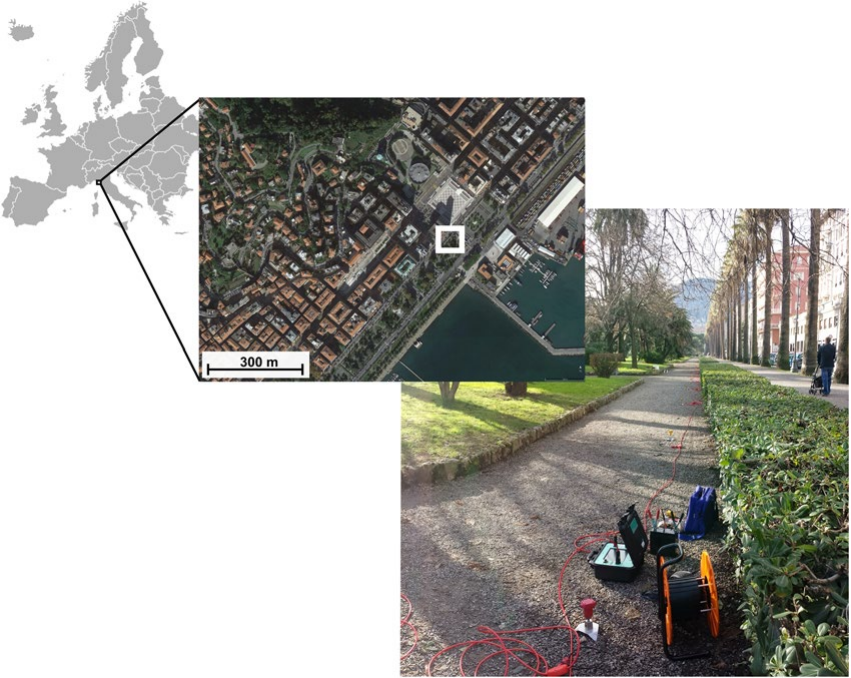
Location of the case study, the city of La Spezia (NW Italy). Satellite image obtained via Google Earth Pro 7.3.2.5776 (https://www.google.com/earth/versions/) (Map data: Image Landsat/Copernicus). The photograph was kindly provided by P3 (Pisa – Italy) together with the seismic field dataset (see *Acknowledgements*).

The considered site is located in a NW-Italy urban area (La Spezia) characterized by about 15 m of soft sediments covering a thick sequence of gravel-like materials^13^. Multi-component (Z, R and T) multi-offset data were acquired along the pathway (covered with a stiff layer of crushed gravel - Fig. 12) of a city park. The acquisition parameters are reported in Table 1 and the data are available for download (see *Data availability statement*).

**Table 1:** Acquisition parameters.

sampling rate	1 ms (1000 Hz)
acquisition length	1 s
minimum offset	5 m
geophone spacing	1 m
number of channels	48
source	8 kg sledgehammer
stack	10

**Figure 13:**
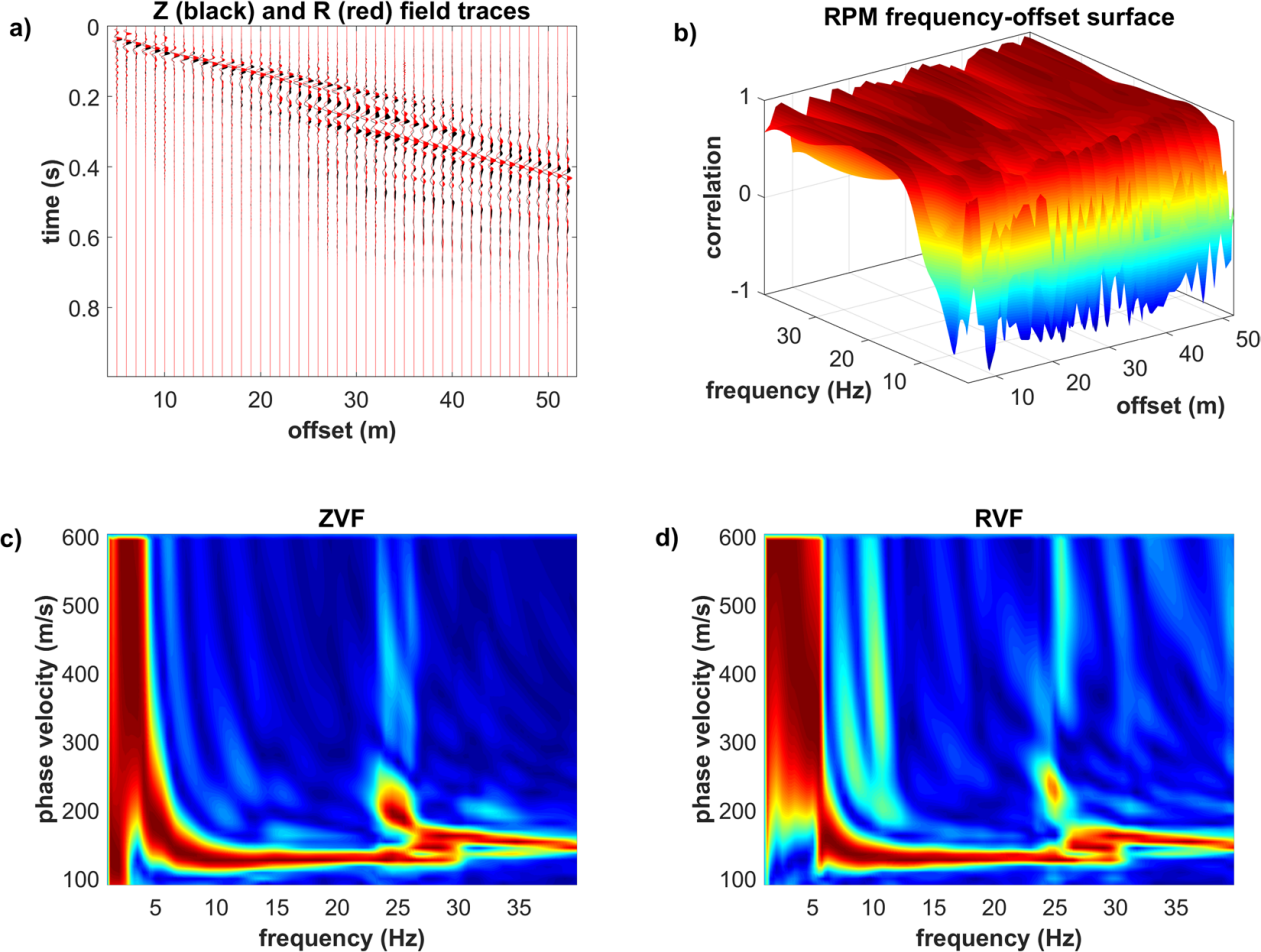
Rayleigh waves: (**a**) field traces of the Z (black) and R (red) components: (**b**) RPM frequency-offset surface; (**c**) phase-velocity spectrum of the Z component; (**d**) phase-velocity spectrum of the R component. See text for comments.

Rayleigh-wave data (phase-velocity spectra of the Z and R components and RPM frequency-offset surface) are shown in Fig. 13. Although at high frequencies (above about 24 Hz) some energy related to higher mode(s) is apparent (see Fig. 13c,d), the overall energy distribution does not show any peculiar characteristic. On the other side, the RPM frequency-offset surface (Fig. 13b) puts in evidence a very distinctive feature: between about 4 and 6 Hz, the particle motion changes from retrograde (correlation value equal to about +1) to prograde (correlation value equal to about −1).

Although the actual Rayleigh wave particle motion is the result of several parameters and it is actually impossible to predict its behaviour (even very simple subsurface models can excite prograde motion) in some cases it was observed that prograde motion is the result of an abrupt increase of the V_S_^13,32,43,44^.

**Figure 14:**
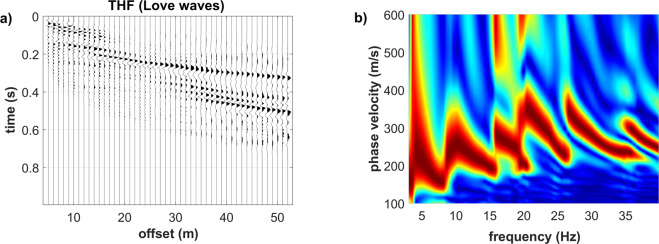
THF component (Love waves): (**a**) field traces; (**b**) phase-velocity spectrum. The top velocity of the higher modes reaches a value of about 350–400 m/s.

Figure 14 reports the field traces and phase-velocity spectrum of the recorded Love waves (THF component). Similarly to the synthetic data presented in the previous sections for the models#2 3 and 4, higher modes appear strongly exited and their top velocities is around 350–400 m/s.

The four computed observables (the phase-velocity spectra of the Z, R and T components and the RPM frequency-offset surface) were jointly analysed according to the multi-objective approach based on the Pareto optimality^27,34–38,52^.

**Figure 15:**
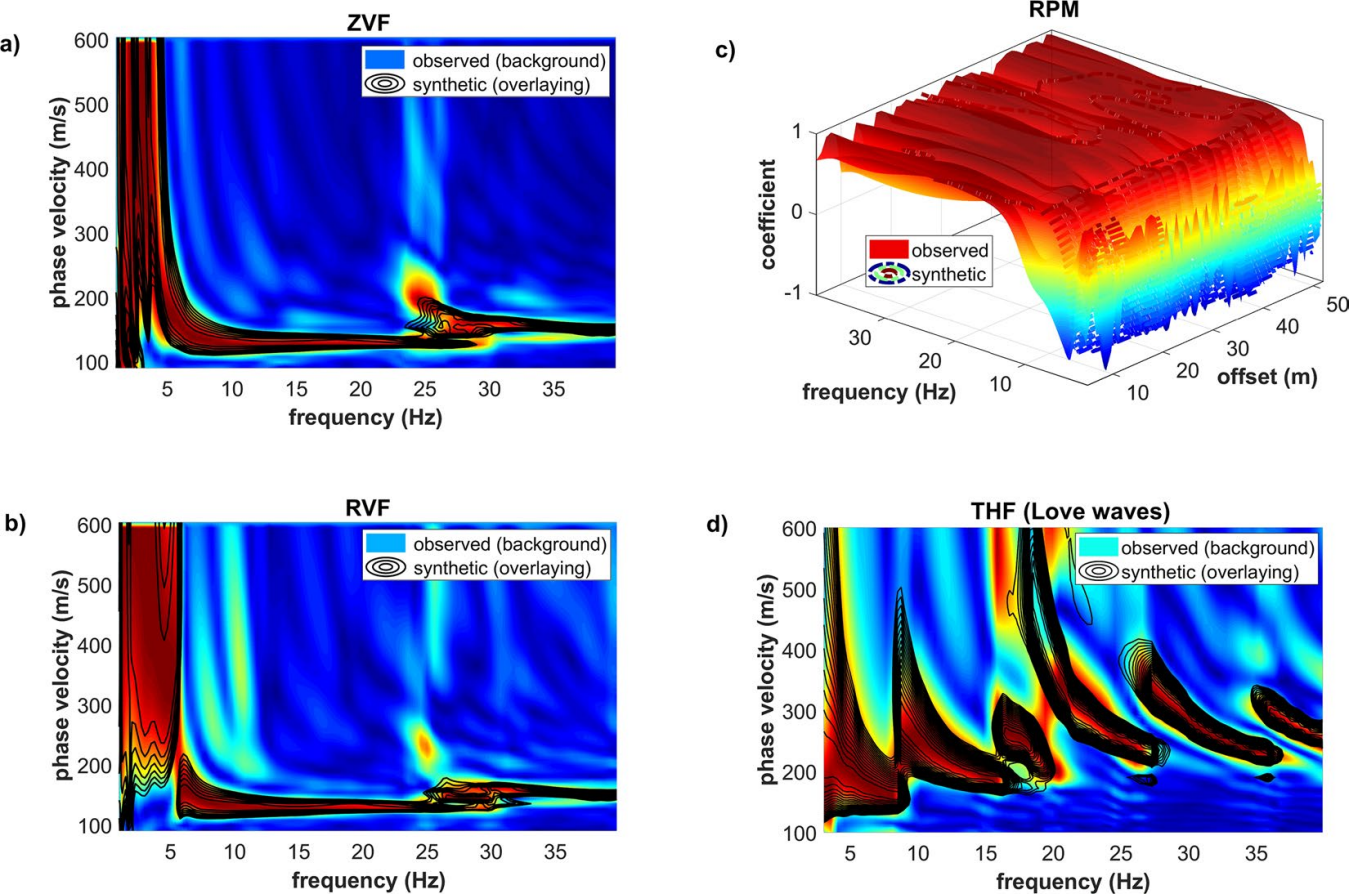
Result of the joint inversion of the four considered observables: (**a**) phase-velocity spectrum of the ZVF component; (**b**) phase-velocity spectrum of the RVF component; (**c**) RPM surface; (**d**) phase-velocity spectrum of the THF component (Love waves). The synthetic data refer to the V_S_ model reported in Fig. 16.

**Figure 16:**
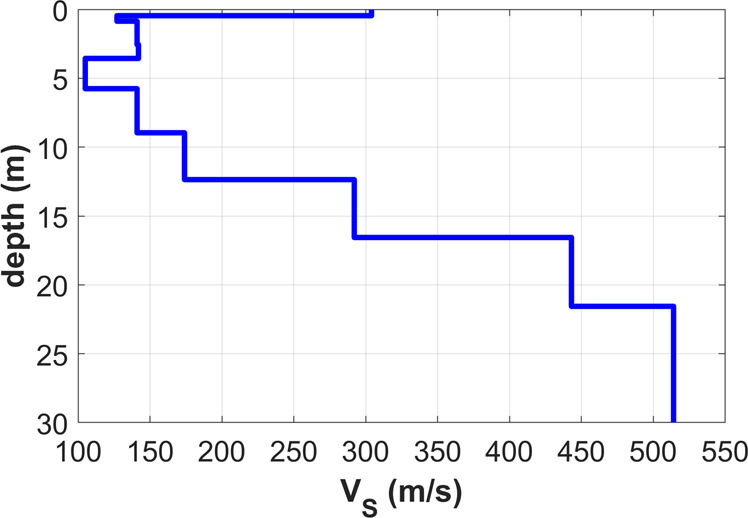
V_S_ model obtained from the joint analysis of the Z, R and T velocity spectra also jointly with the RPM frequency-offset surface (see data and analysis presented in Fig. 15).

The results of the accomplished joint inversion are presented in Figs. 15 and 16 (the shown V_S_ model is the one having the minimum geometrical distance from the utopia point, often referred to as the minimum-distance model^13,19,25,29^).

The effect of the thin superficial stiff layer (the crushed-gravel park pathway) on Love waves is apparent (Fig. 15d): higher modes are excited in the peculiar way already described in the previous sections and their top velocity relates to the shear-wave velocity of the gravels present at a depth of about 15–17m (Fig. 16).

## Conclusions

Through both synthetic and field data, we showed the effect induced by a shallow stiff layer on surface wave propagation. Main facts can be summarized in four points:

1. a shallow stiff layer excites large-amplitude THF (Love waves) higher modes;
2. the top velocity of such Love-wave higher modes is strictly related to the shear-wave velocity of the deeper layer even at very high frequencies and this allows the estimation of the deep V_S_ values even by considering just the high frequencies (these two facts can be summarized with the expression “magnifying effect”);
3. by moving upwards the deep stiff layer, the number of higher modes decreases but the top velocity does not significantly change;
4. the Rayleigh-wave velocity spectra of the Z and R components and the RPM frequency-offset surface (i.e. the particle motion induced by the Rayleigh-wave propagation) are not massively influenced by the presence of a thin shallow stiff layer.

One of the consequences is that, through the *Full Velocity Spectrum* analysis of Love waves, we can define the V_S_ values of the deep layers by considering even just the high frequencies. The FVS approach provides in fact the evidence that a certain mode is actually excited and, consequently, represents a powerful tool that, compared to the standard modal dispersion analysis (which does not provide the proof that a certain mode is actually excited), is capable of better constraining the inversion process.

Needless to say that while the analysis of a single component cannot fully solve possible ambiguities (non-uniqueness of the solution), the joint inversion of the velocity spectra of the Z, R and T components together with the RPM frequency-offset surface is capable of providing an highly-constrained (i.e. robust) subsurface model.

## Data Availability

The field data presented in the “5. A field dataset” section are available from the corresponding author on reasonable request or can be download from the following link: https://doi.org/10.6084/m9.figshare.11913750.

## References

[1] Levshin AL, Pisarenko VF, Pogrebinsky GA (1972). On a Frequency Time Analysis of Oscillations. Ann. Geophys..

[2] Panza Giuliano F. (1981). The Resolving Power of Seismic Surface Waves with Respect to Crust and Upper Mantle Structural Models. The Solution of the Inverse Problem in Geophysical Interpretation.

[3] Levshin AL, Ratnikova LI, Bergher J (1992). Peculiarities of surface wave propagation across Central Eurasia. Bull. Seismol. Soc. Am..

[4] Živčić M, Bondár I, Panza GF (2000). Upper Crustal Velocity Structure in Slovenia from Rayleigh Wave Dispersion. Pure and Applied Geophysics.

[5] Pedersen HA, Bruneton M, Maupin V, The SVEKALAPKO Seismic Tomography Working group (2006). Lithospheric and sublithospheric anisotropy beneath the Baltic shield from surface-wave array analysis. Earth Planet. Sci. Lett..

[6] Fang L, Wu J, Ding Z, Panza GF (2010). High resolution Rayleigh wave group velocity tomography in North China from ambient seismic noise. Geophysical Journal International.

[7] Prodehl C, Kennett B, Artemieva IM, Thybo H (2013). 100 years of seismic research on the Moho. Tectonophysics.

[8] Long L (2001). Surface-wave group-velocity tomography for shallow structures. Journal of Environmental and Engineering Geophysics.

[9] O’Neill A, Dentith M, List R (2003). Full-waveform P-SV reflectivity inversion of surface waves for shallow engineering applications. Exploration Geophysics.

[10] O’Neill A, Matsuoka T (2005). Dominant Higher Surface-wave Modes and Possible Inversion Pitfalls. Journal of Environmental & Engineering Geophysics.

[11] Dou S, Ajo-Franklin JB (2014). Full-wavefield inversion of surface waves for mapping embedded low-velocity zones in permafrost. Geophysics.

[12] Ikeda T, Tsuji T (2016). Surface wave attenuation in the shallow subsurface from multichannel– multishot seismic data: a new approach for detecting fractures and lithological discontinuities. Earth, Planets and Space.

[13] Dal Moro. G (2019). Effective Active and Passive Seismics for the Characterization of Complex Areas: Four Channels for Seven Objective Functions. Pure and Applied Geophysics.

[14] Park, C. B., Xia, J. & Miller, R. D. Imaging dispersion curves of surface waves on multichannel record. In: Proceedings SEG (Society of Exploration Geophysicists) 2003, 68^th^ Annual Meeting, New Orleans, September 13–18 1998, Louisiana, pp. 1377–1380 (1998).

[15] Winsborrow G, Huwsa DG, Muyzertb E (2003). Acquisition and inversion of Love wave data to measure the lateral variability of geo-acoustic properties of marine sediments. Journal of Applied Geophysics.

[16] Natale, M., Nunziata, C. & Panza, G. F. FTAN method for the detailed definition of Vs in urban areas. In: 13^th^ World Conference on Earthquake Engineering, August 1–6, 2004, Vancouver, B.C., Canada, p. 2694 (2004).

[17] Safani J, O’Neill A, Matsuoka T, Sanada Y (2005). Applications of Love Wave Dispersion for Improved Shear-wave Velocity Imaging. Journal of Environmental and Engineering Geophysics.

[18] Dal Moro, G. Surface Wave Analysis for Near Surface Applications, Elsevier, ISBN 9780128007709, 252pp (2014).

[19] Dal Moro G, Moustafa SR, Al-Arifi N (2017). Improved Holistic Analysis of Rayleigh Waves for Single- and Multi-Offset Data: Joint Inversion of Rayleigh-wave Particle Motion and Vertical- and Radial-Component Velocity Spectra. Pure and Applied Geophysics.

[20] Ohori M, Nobata A, Wakamatsu K (2002). A comparison of ESAC and FK methods of estimating phase velocity using arbitrarily shaped microtremor analysis. Bulletin of the Seismological Society of America.

[21] Poggi V, Fäh D (2010). Estimating Rayleigh wave particle motion from three-component array analysis of ambient vibrations. Geophys. J. Int..

[22] Cho I, Senna S, Fujiwara H (2013). Miniature array analysis of microtremors. Geophysics.

[23] Asten MW, Hayashi K (2018). Application of the Spatial Auto-Correlation Method for ShearWave Velocity Studies Using Ambient Noise. Surveys in Geophysics.

[24] Zhang SX, Chan LS (2003). Possible effects of misidentified mode number on Rayleigh wave inversion. J. Appl. Geophys..

[25] Dal Moro G, Moura RM, Moustafa SR (2015). Multi-component Joint Analysis of Surface Waves. J. Appl. Geophysics.

[26] Dal Moro G (2019). Surface wave analysis: improving the accuracy of the shear-wave velocity profile through the efficient joint acquisition and Full Velocity Spectrum (FVS) analysis of Rayleigh and Love waves. Exploration Geophysics.

[27] Dal Moro G, Ferigo F (2011). Joint Analysis of Rayleigh and Love Wave Dispersion for NearSurface Studies: Issues, Criteria and Improvements. J. Appl. Geophysics.

[28] Scales, J. A., Smith, M. L. & Treitel, S. Introductory Geophysical Inverse Theory. open file. Samizdat Press, 193 pp. (2001). open file, http://terra.rice.edu/department/faculty/zelt/esci641/scales/scales.pdf, (accessed April 2020).

[29] Dal Moro G, Puzzilli LM (2017). Single- and Multi-Component Inversion of Rayleigh Waves Acquired by a Single 3-Component Geophone: an Illustrative Case Study. Acta Geodyn. Geomater..

[30] Dal Moro G, Keller L, Poggi V (2015). A Comprehensive Seismic Characterization via MultiComponent Analysis of Active and Passive. Data. First Break.

[31] Dal Moro G (2010). Insights on Surface-Wave Dispersion Curves and HVSR: Joint Analysis via Pareto Optimality. J. Appl. Geophysics.

[32] Dal Moro G, Al-Arifi N, Moustafa SR (2017). Analysis of Rayleigh-Wave Particle Motion from Active Seismics. Bulletin of the Seismological Society of America.

[33] Ritzwoller MH, Levshin AL (2002). Estimating shallow shear velocities with marine multicomponent seismic data. Geophysics.

[34] Van Veldhuizen, D. A. & Lamont, G. B. Evolutionary Computation and Convergence to a Pareto Front. In: Koza, John R. (Ed.), Late Breaking Papers at the Genetic Programming 1998 Conference. Stanford University, pp. 221–228 (1998).

[35] Ramík J, Vlach M (2002). Pareto-optimality of compromise decisions. Fuzzy Sets and Systems.

[36] Deb, K. Multi-Objective Optimization Using Evolutionary Algorithms, John Wiley & Sons, Inc. New York, NY, USA, ISBN:047187339X, 518 pp. (2001).

[37] Pardalos, P.M., Migdalas, A., & Pitsoulis, L. (Eds). Pareto Optimality, Game Theory and Equilibria. Springer, ISBN 978-0-387-77247-9 (2008).

[38] Sawaragi, Y., Nakayama, H. & Tamino, T. Theory of Multiobjective Optimization. Academic Press, Orlando, Florida, 296 pp (1985).

[39] Shtivelman V (2002). Surface wave sections as a tool for imaging subsurface inhomogeneities. Eur. J. Environ. Eng. Geophys..

[40] Safani J., O’Neill A. & Matsuoka T. Love Wave Modelling and Inversion for Low Velocity Layer Cases. Proceedings of the *Symposium on the Application of Geophysics to Eng ineering and Environmental Problems* 2006.

[41] Eslick R, Tsoflias G, Steeples D (2008). Field investigation of Love waves in near-surface seismology. Geophysics.

[42] Levshin AL, Panza GF (2006). Caveats in Multi-modal Inversion of Seismic Surface Wavefields. Pure and Applied Geophysics.

[43] Tanimoto T, Rivera L (2005). Prograde Rayleigh wave motion. Geophys. J. Int..

[44] Malischewsky PG (2008). The domain of existence of prograde Rayleigh-wave particle motion for simple models. Wave Motion.

[45] Herrmann RB (2013). Computer programs in seismology: an evolving tool for instruction and research. Seismological Research Letters.

[46] Herrmann, R. B. Computer Programs in Seismology. Open files (http://www.eas.slu.edu/People/RBHerrmann/CPS330.html), (accessed April 2020).

[47] Panza GF (1985). Synthetic seismograms: the Rayleigh waves modal summation. J. Geophys..

[48] Panza, G. F. Attenuation measurements by multimode synthetic seismograms, In: *Digital seismology and fine modeling of the Lithospehere*, Editors: R. Cassinis, G. Nolet and G.F. Panza, Plenum Publishing Corporation, 79–115 (1989).

[49] Panza GF, Romanelli F, Vaccari F (2001). Seismic wave propagation in laterally heterogeneous anelastic media: theory and applications to seismic zonation. Advances in Geophysics.

[50] Lunedei E, Albarello D (2009). On the seismic noise wavefield in a weakly dissipative layered Earth. Geophys. J. Int..

[51] Beaty KS, Schmitt DR, Sacchi M (2002). Simulated annealing inversion of multimode Rayleigh wave dispersion curves for geological structure. Geophysical Journal International.

[52] Dal Moro G, Pipan M (2007). Joint inversion of surface wave dispersion curves and reflection travel times via multi-objective evolutionary algorithms. J. Appl. Geophys..

